# Identification of bovine CpG SNPs as potential targets for epigenetic regulation via DNA methylation

**DOI:** 10.1371/journal.pone.0222329

**Published:** 2019-09-12

**Authors:** Mariângela B. C. Maldonado, Nelson B. de Rezende Neto, Sheila T. Nagamatsu, Marcelo F. Carazzolle, Jesse L. Hoff, Lynsey K. Whitacre, Robert D. Schnabel, Susanta K. Behura, Stephanie D. McKay, Jeremy F. Taylor, Flavia L. Lopes

**Affiliations:** 1 São Paulo State University (Unesp), School of Veterinary Medicine, Araçatuba, São Paulo, Brazil; 2 Natural and Human Sciences Center, ABC Federal University, Santo André, São Paulo, Brazil; 3 Genomics and Expression Laboratory, University of Campinas, Campinas, São Paulo, Brazil; 4 Brazilian Bioethanol Science and Technology Laboratory (CTBE), Brazilian Center for Research in Energy and Materials (CNPEM), Campinas, São Paulo, Brazil; 5 National Center for High Performance Computing (CENAPAD-SP), University of Campinas, Campinas, São Paulo, Brazil; 6 Division of Animal Sciences, University of Missouri, Columbia, Missouri, United States of America; 7 Informatics Institute, University of Missouri, Columbia, Missouri, United States of America; 8 Department of Animal and Veterinary Sciences, University of Vermont, Burlington, Vermont, United States of America; National Institutes of Health, UNITED STATES

## Abstract

Methylation patterns established and maintained at CpG sites may be altered by single nucleotide polymorphisms (SNPs) within these sites and may affect the regulation of nearby genes. Our aims were to: 1) identify and generate a database of SNPs potentially subject to epigenetic control by DNA methylation via their involvement in creating, removing or displacing CpG sites (meSNPs), and; 2) investigate the association of these meSNPs with CpG islands (CGIs), and with methylation profiles of DNA extracted from tissues from cattle with divergent feed efficiencies detected using MIRA-Seq. Using the variant annotation for 56,969,697 SNPs identified in Run5 of the 1000 Bull Genomes Project and the UMD3.1.1 bovine reference genome sequence assembly, we identified and classified 12,836,763 meSNPs according to the nature of variation created at CpGs. The majority of the meSNPs were located in intergenic regions (68%) or introns (26.3%). We found an enrichment (p<0.01) of meSNPs located in CGIs relative to the genome as a whole, and also in differentially methylated sequences in tissues from animals divergent for feed efficiency. Seven meSNPs, located in differentially methylated regions, were fixed for methylation site creating (MSC) or destroying (MSD) alleles in the differentially methylated genomic sequences of animals differing in feed efficiency. These meSNPs may be mechanistically responsible for creating or deleting methylation targets responsible for the differential expression of genes underlying differences in feed efficiency. Our methyl SNP database (dbmeSNP) is useful for identifying potentially functional "epigenetic polymorphisms" underlying variation in bovine phenotypes.

## Introduction

Epigenetic events regulate gene expression through potentially transient changes to the chromatin without actually altering the nucleotide sequence, allowing genetically identical cells to differentiate phenotypically within and between cell lineages [1]. Such epigenetic mechanisms include DNA methylation, histone remodeling and DNA or mRNA interactions with non-coding RNAs.

DNA methylation, primarily characterized by the addition of a methyl group at the 5-position of the cytosine pyrimidine ring in CG dinucleotides, is a fundamental epigenetic modification that occurs in many cellular processes, such as the development and maintenance of chromatin structure, parental imprinting, and X chromosome inactivation in females [2–4], and has an important role in the regulation of gene expression [5]. The loss of methylation patterns in murine embryos is lethal, demonstrating the vital role of this epigenetic mechanism to the development [6] of organisms.

Chromatin activity and DNA methylation status are highly correlated [7], with the presence of methylation generally resulting in the silencing of gene expression [8]. Conversely, DNA hypomethylation is generally associated with active transcription. Recent studies have associated single nucleotide polymorphisms (SNPs) with differential DNA methylation and these changes in methylation patterns lead to variation in the expression of nearby genes [9–11]. However, the association between genetic variation and DNA methylation and the genetic determinants of DNA methylation patterns are unclear [10–13]. Genetic variation at cytosine-phosphate-guanine (CpG) sites can disrupt methylation sites and, therefore, drastically change the methylation state [14,15]. The introduction or removal of a CpG site, potentially subject to DNA methylation, has been suggested as a mechanism by which SNPs can affect gene regulation through altered epigenetic patterns [9].

SNPs are important markers that have been used to associate specific genomic regions to normal physiological changes, diseases, response to pathogens, chemicals, drugs, and vaccines in humans [16,17]. SNP studies have also been important in the development and enhancement of breeding programs in animals and plants, and high density genotype information has been used to locate quantitative trait loci (QTL), identify chromosomal regions exposed to strong selection, elucidate the evolutionary history of populations, and characterize/manage genetic resources and diversity [18,19].

Since modifications caused by SNPs at CpG sites may potentially be associated with changes in the expression of nearby genes and, consequently, with phenotype determination, we sought to: 1) identify SNPs that are potential targets for epigenetic control via by DNA methylation, through their involvement in the creation, removal or displacement of CpG sites (meSNPs); 2) evaluate the association of these meSNPs with CpG islands (CGIs), using the genome coordinates of predicted CGIs for bovine available on the National Center for Biotechnology Information (NCBI) website; 3) investigate the relationship of alleles at these meSNPs with methylation profiles found in liver, *longissimus dorsi* and small intestine, determined using the methylated-CpG island recovery assay combined with next generation sequencing (MIRA-Seq) in cattle; and 4) determine if the meSNPs present in differentially methylated regions (DMRs) associated with high and low feed efficiency are located in QTL regions for this trait. To accomplish these objectives, we created a novel database including functional annotations for meSNPs in which we associated the meSNPs with CGIs and methylated DNA fractions in bovine tissues. Our methyl SNP database (dbmeSNP) (https://dbmesnp.wixsite.com/epigenetics) can be used to associate genetic variation with DNA methylation patterns and bovine traits.

## Materials and methods

### Ethics statement

This study was conducted with an approved animal use protocol from the Institutional Animal Care and Use Committee at the University of Missouri (7505).

### Compatibility between 1000BGP Run 5 and the UMD3.1.1 reference genome

To confirm the compatibility between the annotated SNPs identified in Run5 of the 1000 Bull Genomes Project (1000BGP) [20] and the UMD3.1.1 bovine reference genome assembly downloaded from the NCBI database [21] (https://www.ncbi.nlm.nih.gov/genome/?term=Bos_taurus_UMD_3.1.1), we developed a python script (2.7.12 version, pattern Nov 19 2016) to verify: 1) the reference base for each SNP annotated in the 1000BGP, 2) chromosome number, and 3) SNP position. Insertion/deletion markers (INDELs) identified in the 1000BGP were not considered in this study.

### Mapping of SNPs to CpG sites

**Fig 1:**
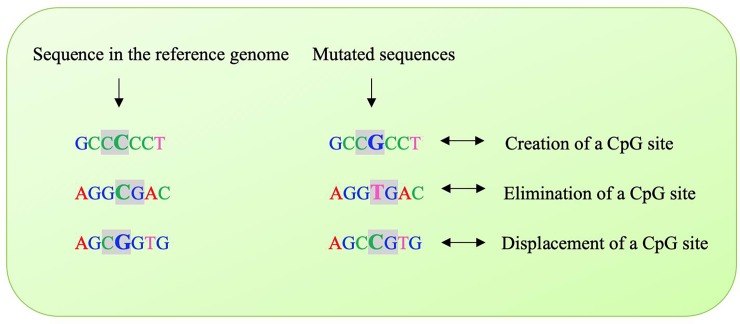
Examples of variants caused by meSNP. The modification caused by a meSNP can create, destroy or displace a CpG site, the disruption of these sites can drastically change the methylation state. Base in bold represents a meSNP.

To map SNPs to CpG sites, which are potentially subject to methylation, we created python scripts to retrieve the flanking nucleotides in the reference genome for each 1000BGP Run5 SNP and to classified meSNPs, genetic variants at CpG sites [11], according to whether they: 1) created a CpG site; 2) destroyed a CpG site; or 3) displaced a CpG site (Fig 1). Genomic coordinates of each SNP were based on the forward strand of the UMD3.1.1 reference genome.

### Association between meSNPs and their variant functional annotations

The functional implications of the genomic variants at each meSNP were evaluated using Ensembl's Variant Effect Predictor (VEP) [22]. Using a perl script (v5.10.1, Copyright 1987–2009, Larry Wall) we integrated information contained in the Variant Call Format output file generated by VEP with the meSNPs identified among the Run5 1000BGP SNPs. This script created, for each chromosome, files containing the meSNP information along with information extracted from the Variant Call Format file for meSNPs. We also classified the meSNPs into 13 different categories based on the 35 sequence ontology (SO; http://www.ensembl.org/info/genome/variation/predicted_data.html) terms, as described in S1 Table.

### Identification of meSNPs in CGIs

Bovine CGI annotations were extracted from NCBI [21] (ftp://ftp.ncbi.nlm.nih.gov/genomes/MapView/Bos_taurus/sequence/BUILD.6.1/initial_release/). Two sets of criteria were used to identify strict and relaxed CGIs, where relaxed CGIs are ≥ 200 bp in length, and strict CGIs are ≥ 500 bp in length; and both possess ≥ 50% G + C content and have ≥ 0.60 observed CpG/expected CpG, as described in https://www.ncbi.nlm.nih.gov/projects/mapview/static/cowsearch.html#cpg. Data processing was performed separately for each criterion. Annotations for the UMD3.1 assembly were considered. The difference between the UMD3.1.1 assembly and its predecessor, UMD3.1, is the elimination of 173 contigs, however, the chromosomal coordinate system is identical between both assemblies [23]. MeSNPs located within strict or relaxed CGIs were mapped by means of a python script.

### DNA extraction and MIRA-Seq

DNA was extracted according to methods previously described [24] from liver, *longissimus dorsi* and small intestine tissues from each of eight Angus steers differing in feed efficiency (four high feed efficiency and four low feed efficiency), and fed as a cohort with a total of 96 contemporary animals at the Circle A Ranch (Huntsville, Stockton and Iberia, MO, USA). MIRA-Seq libraries were generated from the DNA from each tissue of each animal. Initially, 1.5 ug of DNA was sonicated to 200 to 500 bp fragments using a Bioruptor standard (Diagenode, Danville, NJ). For purification of the DNA fragments we used the Qiagen MinElute PCR Purification kit and sonication accuracy was visualized by electrophoresis with a 1% agarose gel stained with SYBR green. For library construction, we used the NEB Next DNA Library Prep Master Mix Set for Illumina kit (New England BioLabs, Ipswich, MA) according to manufacturer’s instructions with some modifications, as previously described [25,26]. Briefly, sonicated and purified DNA underwent end-repair, followed by another round of purification prior to dA-tailing using the NEBNext DNA Library kit, following manufacturer’s instructions. After cleanup, adaptors were ligated onto the DNA fragments.

For library construction, custom paired-end barcoded adaptors were ligated to dA-tailed DNA, according to manufacturer’s recommendations with one modification, where 1 ul of 50 uM adaptors was utilized for library construction. Adaptor ligated dA-tailed DNA was purified with the Qiagen MinElute PCR Purification kit.

Methyl Collector Ultra Kit (Active Motif, Carlsbad, CA) was used for MIRA pulldown, according to manufacturer’s instructions. Size selection of adaptor-ligated methylated DNA fragments, and removal of excess adaptors, were performed using the Qiagen MinElute Gel Extraction kit. Library construction concluded with PCR enrichment. Each reaction consisted of 20 ng of DNA, 1 ul of each of two custom universal primers, 25 ul of Phusion® High-Fidelity PCR Master Mix with GC Buffer (New England BioLabs, Ipswich, MA) and nuclease-free water to yield a total volume of 50 ul, under the following cycling conditions: denaturation at 98°C for 30 seconds; 12 cycles of denaturation at 98°C for 30 seconds, annealing at 65°C for 30 seconds and extension at 72°C degrees for 30 seconds followed by a final extension of 72°C for 5 minutes. Each library underwent gel purification using the Qiagen MinElute Gel Extraction kit to remove excess primers. Confirmation of enrichment for each library was performed using PCR primers designed to amplify a known methylated region of *LIT1*, and a non-methylated region of *MP68*. Primers and PCR conditions have been previously reported [26]. Constructed libraries were submitted to the University of Missouri’s DNA Core facility for sequencing on an Illumina Hi-Seq 2000. Samples were multiplexed (two samples per lane) and sequenced, generating a total of 687.5 Million 100 bp single-end sequence reads.

### Alignment of reads and peak identification

Adaptor sequences were trimmed from sequence reads using a custom perl script [27] and subsequent quality trimming of the sequence reads was performed using NextGENe software version 2.3.3 (SoftGenetics, State College, PA). Sequence reads with a median quality score below 20, 3 or more uncalled bases, or smaller than 35 bp following trimming were removed. The reads were then mapped to the reference genome UMD3.1 [28] using Bowtie2 [29].

### Identification of differentially methylated regions

Analysis of MIRA-Seq data was performed using MACS2 (version 2.1.1.20160309), to identify peaks relative to genome-wide background in the high feed efficiency and low feed efficiency animals, independently. Then, the detected peaks were intersected based on genomic position of significant peaks from both groups using Bedtools to identify whether a peak was present in at least 3 of the 4 animals from each of the groups, and absent in all animals from the other group. An additional filtering step was applied based on peak length, pileup height, and fold enrichment scores generated from the MACS2 outputs. Using this approach, hypermethylated sites were identified that were specific to either the high or low feed efficiency groups.

To identify DMRs, a paired generalized linear model likelihood-ratio test was employed, followed by Benjamini-Hochberg adjustment of raw p-values, to account for multiple comparisons [30]. A region was considered to be differentially methylated when the test statistic achieved a FDR <0.05. Functional annotation of differentially methylated regions was conducted using PANTHER [31].

### Identification of meSNPs in tissues submitted to MIRA-Seq

The meSNPs located within the DMRs detected in each tissue of eight Angus steers using MIRA-Seq were mapped using a python script, executed separately for each tissue. We also quantified and classified the meSNPs separately, according to the variation they caused at the CpG sites for both tissues.

### Statistical analysis to determine meSNP enrichment

We considered the number of meSNPs found on each chromosome, the cumulative length of the DMRs per chromosome and the length of each chromosome in base pairs obtained from https://ccb.jhu.edu/bos_taurus_assembly.shtml. We estimated the expected number of meSNPs that should be found on each chromosome within strict CGIs, relaxed CGIs or in tissues processed by MIRA-Seq based upon a random distribution of sites throughout the genome and ignoring nucleotide composition and tested the statistical difference between the observed and expected meSNP numbers using a Chi-square distribution with a significance level of 1%.

### Association between meSNP alleles in DMRs in tissues from animals with divergent feed efficiency phenotypes

We identified meSNPs within DMRs with genotypes scored in at least 3 of the 4 animals from each feed efficiency group in liver, *longissimus dorsi* and small intestine. To identify which of these meSNPs were also annotated in the Variant Call File (VCF) obtained after genotyping the animals with divergent feed efficiency phenotypes we made a merge between the positions of these meSNPs with positions of the variant annotations for each chromosome and each tissue described in the VCF using a python script. Then, we were able to identify those meSNPs that were concordant with the feed efficiency phenotype. To identify the meSNPs with concordant genotypes in at least 3 of the 4 genotyped animals within each of the high feed efficiency (HFE) or low feed efficiency (LFE) phenotype groups, we calculated the allele frequency (AF) for each meSNP in each tissue using the equation: $AF=AF_{\left(HFE\right)}-AF_{\left(LFE\right)}\;where,\;AF_{\left(HFE\right)}=\frac{sum\;of\;alternate\;allele\;in\;HFE}{total\;number\;of\;allele\;in\;HFE}\;and\;AF_{\left(LFE\right)}=\frac{sum\;alternate\;allele\;in\;LFE}{total\;number\;of\;allele\;in\;LFE}$ and alternate allele refers to the non-reference allele at each meSNP.

In the VCF, the genotypes are listed for each variant in each animal and in each tissue as allele/allele where ./. represents missing genotype; 0/0 represents reference/reference; 0/1 represents reference/alternate, and 1/1 represents alternate/alternate. Consequently, there are 256 possible genotypic combinations for the 4 animals within each group and, of these, there are 245 combinations where 3 of the 4 animals have a valid genotype. To illustrate the AF calculation, consider genotypes 0/0 0/0 0/0 ./. in the HFE and 0/1 0/1 0/1 ./. in the LFE group, leading to an allele frequency difference for the alternate allele of $AF=\left(\frac{3}{6}-\frac{0}{6}\right)=0.5$ or for the genotypes 0/0 0/0 0/0 0/1 in the LFE group and 1/1 1/0 1/1 1/1 in the HFE group the allele frequency difference is $AF=(\frac{7}{8}-\frac{1}{8})=0.75.$ We required a difference in the absolute value of AF between the two groups of ≥ 0.5 to declare an association with feed efficiency phenotypes.

Following the identification of candidate meSNPs, we next filtered to retain only those with DNA methylation signals that were compatible with the alteration that they caused at the CpG site, that is, the MSC allele should be at a higher frequency in the hypermethylated group, and the MSD allele should be at a higher frequency in the hypomethylated group.

### Identification of meSNPs associated with the feed efficiency phenotype located within Quantitative Trait Loci (QTL)

The Cattle Quantitative Trait Locus (QTL) Database (Cattle QTLdb) from the National Animal Genome Research Program [32] (https://www.animalgenome.org/cgi-bin/QTLdb/BT/traitsrch?tword=feed%20efficiency) was queried to identify curated cattle QTLs and association data from published data. Cattle QTLdb contains only 35 QTLs associated with feed conversion efficiency. These QTLs were identified for 4 different trait definitions (efficiency of gain, feed efficiency, maintenance efficiency and partial efficiency of growth). The genomic coordinates of the meSNPs with concordant allele associations within DMRs found for the high or low feed efficiency groups were queried against the locations of feed efficiency QTLs identified in Cattle QTLdb.

### Data records

Data produced by MIRA-Seq were deposited with a BioProject record (PRJNA560026). Samples used in the study are shown in S2 Table.

## Results

### Compatibility between Run5 of the 1000 Bull Genomes Project and UMD3.1.1

To conduct the genome-wide meSNP discovery analysis we used the SNP variants annotated in Run5 of the 1000BGP [20]. We first confirmed that the reference nucleotide base and the position annotated for each SNP in UMD3.1.1, was completely consistent with the variant SNP annotations identified in Run5 of the 1000BGP. INDELs were not considered in this study because of a concern regarding the reliability of their identification within Run5 of the 1000BGP.

### Identification and characterization of meSNPs according to the type of variant created at each CpG site

**Fig 2:**
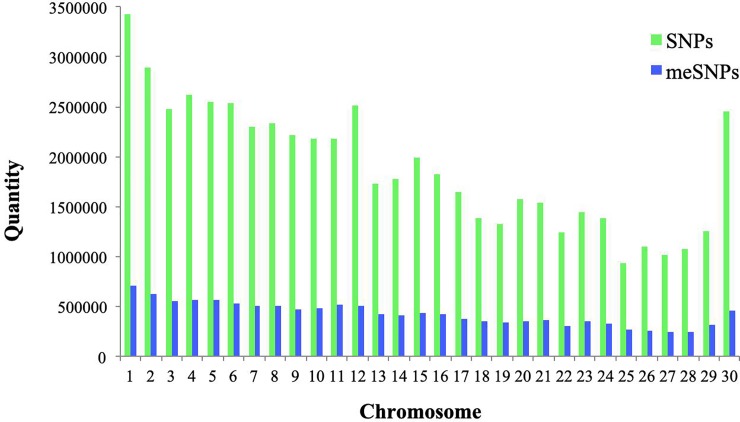
SNPs versus meSNPs. Number of meSNPs identified in comparison to the total number of SNPs annotated in Run5 of the 1000 Bull Genomes Project by chromosome.

We identified and annotated 12,836,763 meSNPs representing 22.53% of the 56,969,697 SNPs annotated in Run5 of the 1000BGP. We also determined the number of meSNPs present on each chromosome (Fig 2). Due to the possible impact of a genome sequence change caused by a meSNP, and the consequence of this change for the potential regulation of gene expression for nearby genes, we classified the meSNPs according to the variation they created at the CpG sites by chromosome (Table 1).

**Table 1:** Characterization of meSNPs according to the variant pattern created at a CpG site.

Chromosome	Creation of a CpG site	%	Elimination of a CpG site	%	Displacement of a CpG site	%
1	331,266	2.58	374,131	2.91	5,165	0.04
2	286,089	2.23	332,345	2.59	5,100	0.04
3	248,693	1.94	297,706	2.32	5,004	0.04
4	260,690	2.03	305,178	2.38	4,707	0.04
5	255,625	1.99	306,213	2.39	5,097	0.04
6	243,815	1.90	279,834	2.18	3,993	0.03
7	228,011	1.78	270,713	2.11	4,393	0.03
8	231,166	1.80	271,667	2.12	4,126	0.03
9	215,600	1.68	256,171	2.00	3,815	0.03
10	218,695	1.70	261,782	2.04	4,094	0.03
11	224,534	1.75	285,744	2.23	5,249	0.04
12	244,606	1.91	257,742	2.01	3,795	0.03
13	184,502	1.44	242,025	1.89	4,339	0.03
14	182,869	1.42	228,305	1.78	3,802	0.03
15	199,706	1.56	235,545	1.83	3,733	0.03
16	187,079	1.46	233,294	1.82	3,999	0.03
17	165,861	1.29	204,661	1.59	3,654	0.03
18	149,413	1.16	199,793	1.56	4,404	0.03
19	142,988	1.11	196,753	1.53	4,674	0.04
20	157,402	1.23	190,181	1.48	2,868	0.02
21	162,161	1.26	205,731	1.60	3,790	0.03
22	130,358	1.02	173,493	1.35	3,066	0.02
23	154,706	1.21	192,864	1.50	3,721	0.03
24	144,810	1.13	186,311	1.45	3,156	0.02
25	106,921	0.83	157,543	1.23	3,491	0.03
26	113,731	0.89	147,737	1.15	2,566	0.02
27	106,800	0.83	138,189	1.08	2,470	0.02
28	112,764	0.88	134,260	1.05	2,116	0.02
29	135,654	1.06	175,098	1.36	3,231	0.03
X	211,563	1.65	240,603	1.87	3,455	0.03
**Total**	**5,738,078**	**44.70**	**6,981,612**	**54.39**	**117,073**	**0.91**

### Functional annotations of meSNPs

**Fig 3:**
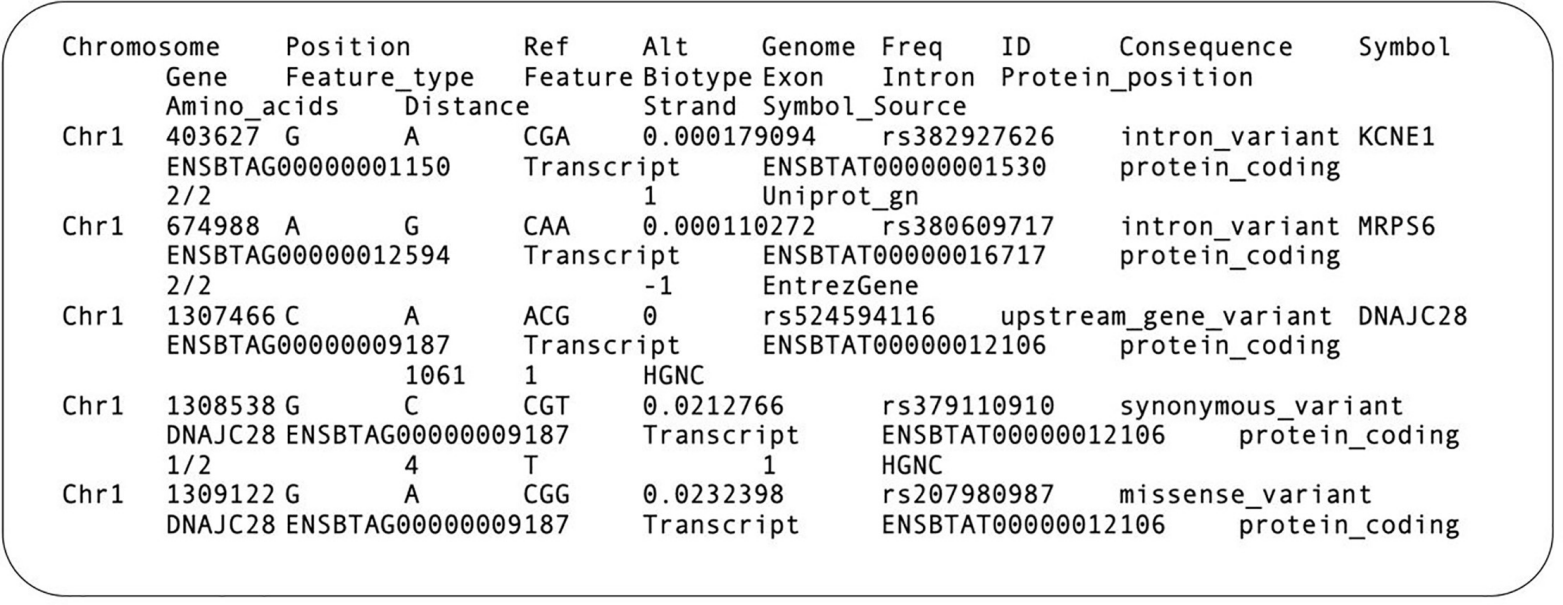
Example of meSNP database conformation, developed from the annotations contained in Run5 of the 1000 Bull Genomes Project and the Variant Effect Predictor. In the "Genome" column the second nucleotide represents the position of the meSNP in reference genome UMD3.1.1, described in the "Position" column; this nucleotide is also described in the "Ref" column. The "Alt" column represents the change caused by meSNP in the genomic sequence.

SNPs involved in the creation, removal or displacement of CpG sites were functionally annotated in the process of developing a bovine meSNP database. These functional annotations were generated using VEP software, and the meSNP database includes information regarding UMD3.1 chromosome number, position, reference allele, alternative allele, 1000BGP allele frequency, rs ID, variant consequence, associated gene, functionality and transcript biotype or regulatory function. This process added 14 functional annotations, not provided in Run5 of the 1000BGP, based on information contained in the Ensembl and NCBI Reference Sequence (RefSeq) databases, employed by VEP. An example of the dbmeSNP information for 5 meSNPs annotated on chromosome 1 is shown in Fig 3.

### Classification of meSNPs according to sequence ontology

**Fig 4:**
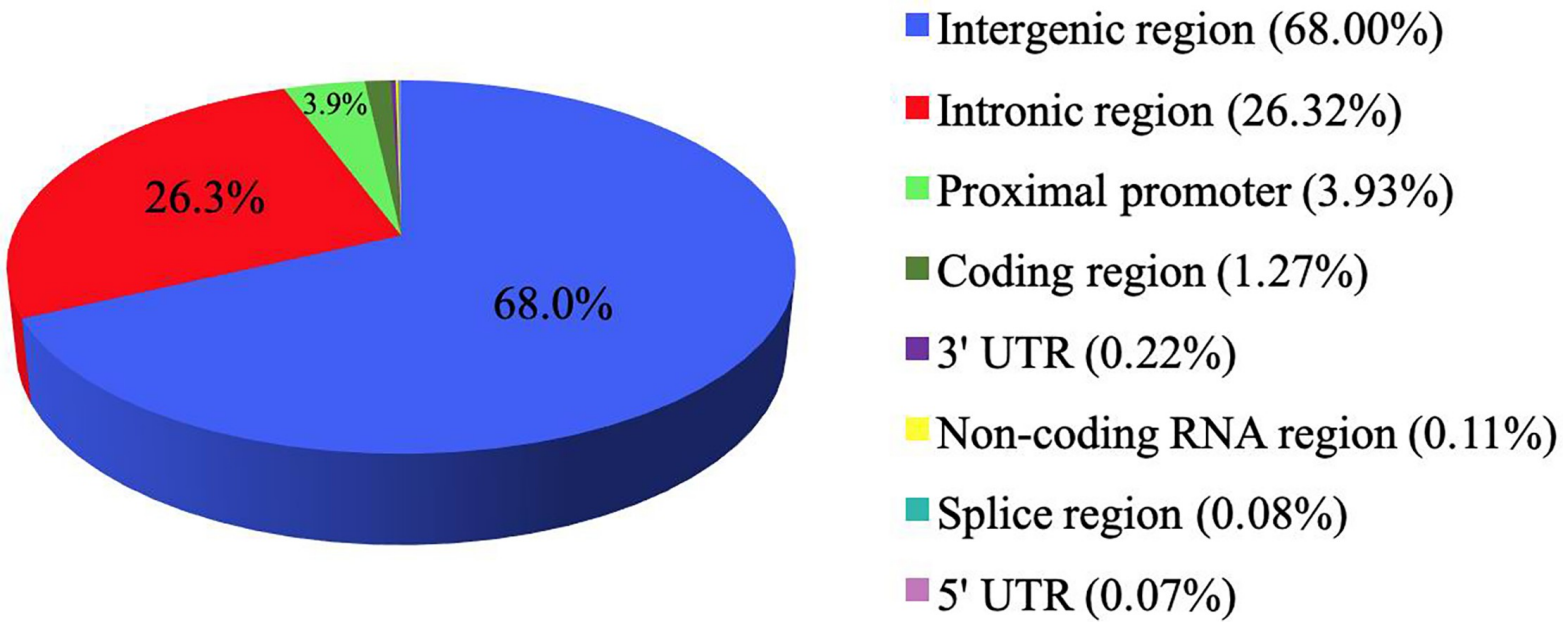
Genomic distribution of meSNPs. Classification of meSNPs according to sequence ontology (SO term) provided by Ensembl.

The vast majority of meSNPs were located in intergenic and intronic regions (>90%), whereas, the remaining meSNPs were distributed between proximal promoters and translated coding regions (~5%), and a small portion were in 5' untranslated regions (UTRs) (0.07%), 3' UTRs (0.22%), non-coding RNAs (0.11%) or splice sites (0.08%) (Fig 4).

### meSNPs in CGIs

**Fig 5:**
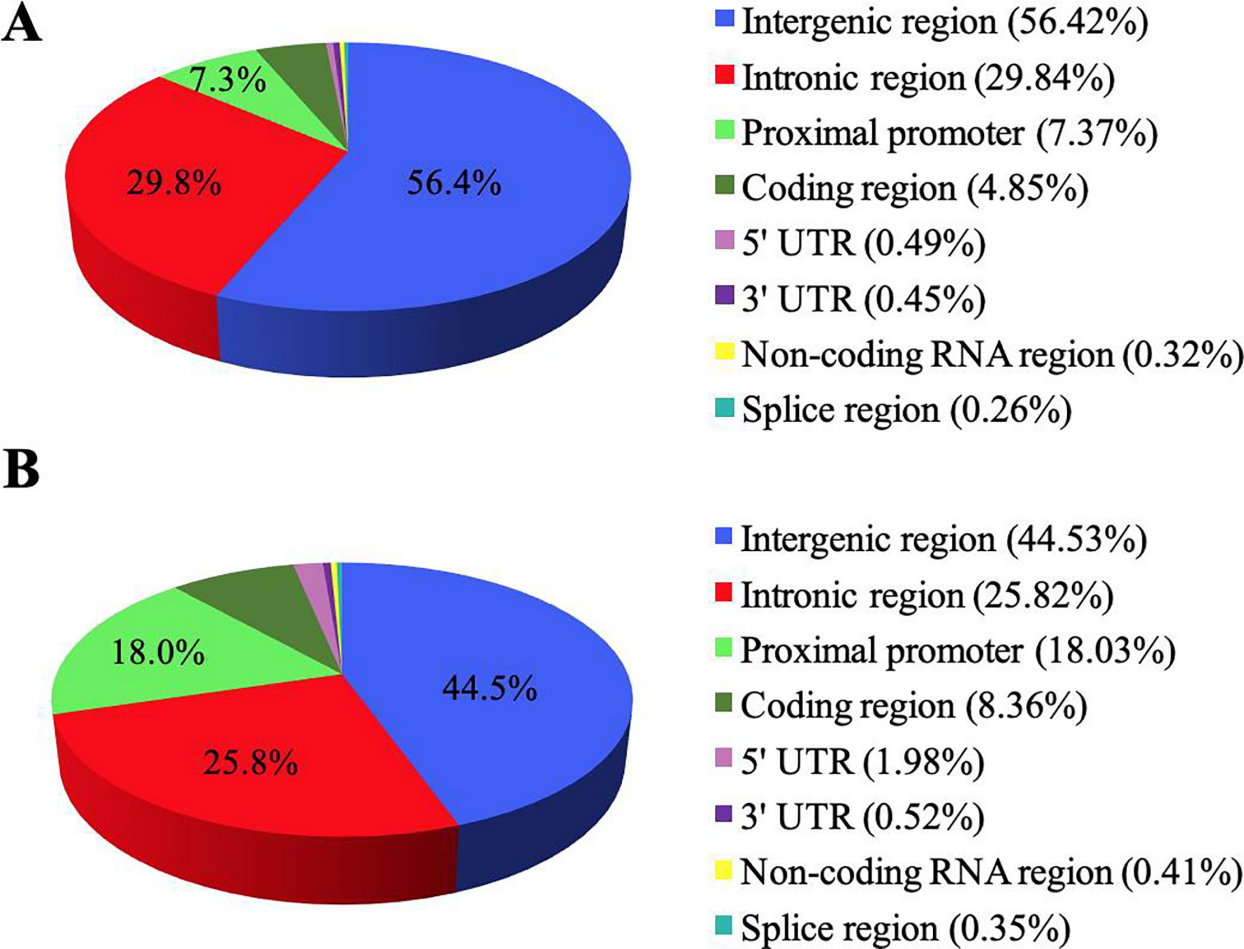
Classification of meSNPs located within CGIs. Genomic distribution of meSNPs identified in (A) relaxed, and (B) strictly defined CGIs.

Only 12.35% of the meSNPs were predicted to be located in CGIs, the majority (10.36%) were found in relaxed CGIs and the remainder in strict CGIs. For both classifications of CGIs, most methyl-markers were found in intergenic, intronic and proximal promoter regions (Fig 5), similarly to the data previously shown in Fig 4, when we considered the genome as a whole rather than just CGIs. The distribution of the meSNPs located in CGIs by chromosomes is provided in Table 2. There was an average enrichment of 2.47 times more meSNPs than expected (p<0.01) within relaxed CGIs based on the proportion of the genome spanned by relaxed CGIs, and for the strict CGIs the average enrichment was 1.90 times greater than expected (p<0.01). The enrichment of meSNPs in relaxed and strict CGIs by chromosome is shown in S3 Table.

**Table 2:** Distribution of meSNPs identified within CGIs by chromosome.

Chromosome	meSNPs in relaxed CGIs	%	meSNPs in strict CGIs	%
1	50,386	0.39	8,127	0.06
2	52,508	0.41	9,358	0.07
3	53,945	0.42	10,997	0.09
4	52,517	0.41	10,679	0.08
5	58,389	0.45	10,191	0.08
6	45,406	0.35	8,085	0.06
7	47,065	0.37	10,957	0.09
8	45,373	0.35	10,589	0.08
9	47,077	0.37	9,478	0.07
10	41,654	0.32	8,846	0.07
11	59,271	0.46	11,541	0.09
12	44,811	0.35	7,172	0.06
13	46,969	0.37	7,654	0.06
14	42,020	0.33	7,563	0.06
15	38,450	0.30	6,501	0.05
16	43,487	0.34	7,346	0.06
17	43,153	0.34	8,406	0.07
18	51,040	0.40	11,114	0.09
19	51,814	0.40	11,535	0.09
20	31,778	0.25	4,967	0.04
21	42,513	0.33	7,902	0.06
22	41,210	0.32	7,303	0.06
23	47,656	0.37	9,448	0.07
24	38,240	0.30	6,706	0.05
25	49,157	0.38	11,502	0.09
26	32,560	0.25	5,966	0.05
27	32,810	0.26	6,292	0.05
28	20,847	0.16	4,550	0.04
29	40,902	0.32	7,506	0.06
X	37,062	0.29	7,615	0.06
**Total**	**1,330,070**	**10.36**	**255,896**	**1.99**

### meSNPs in differentially methylated regions in tissues of animals differing for feed efficiency

Samples of liver, *longissimus dorsi* and small intestine from 4 Angus steers with low feed efficiency and 4 with high feed efficiency were processed by MIRA-Seq, resulting in sequence reads with an average mapping percentage of 98.24% for liver, 91.57% for *longissimus dorsi* and 98.33% for small intestine. Peak calls were performed using MACS2 to identify peaks relative to genome-wide background in the high feed efficiency and low feed efficiency groups independently, which detected 21,967 DMRs in liver, 29,597 DMRs in *longissimus dorsi* and 55,172 DMRs in small intestine.

Of the 86,510 meSNPs within the 21,967 DMRs predicted in liver, 71.32% destroyed CpG sites, 26.37% created new CpG sites and 2.31% caused the displacement of the CpG site. In the *longissimus dorsi*, of the 137,818 meSNPs within 29,597 predicted DMRs, 70.25% destroyed CpG sites, 27.59% created new CpG sites and 2.16% caused the displacement of the CpG site. In the small intestine, of the 206,106 meSNPs within 55,172 predicted DMRs, 72.41% destroyed CpG sites, 25.25% created new CpG sites and 2.34% caused the displacement of the CpG site. While these frequencies appear quite similar, the sample size is sufficient that they differ statistically (p<0.01).

In liver, we found an average enrichment of 2.83 and 3.36 times more meSNPs than expected (p<0.01) for high and low feed efficiency animals, respectively; for *longissimus dorsi*, the average enrichment was 2.87 and 2.82 times greater than expected (p<0.01) for high and low feed efficiency animals, respectively; and for small intestine the average enrichment was 2.72 and 2.89 times greater than expected (p<0.01) for high and low feed efficiency animals, respectively based on the proportion of the genome represented in these DMRs. Enrichment of meSNPs in DMRs in the tissues submitted to MIRA-Seq, by chromosome, is shown in S4 Table.

### Allelic associations for meSNPs in differentially methylated regions

After independently identifying the peaks for each sample, the results were intersected based on genomic position using Bedtools to identify DMRs that were detected in at least 3 of the 4 animals within each feed efficiency group, indicating possible feed efficiency specific methylation patterns in each tissue. For allelic association analysis, we used meSNPs that were located within these DMRs. Next, we made an attempt to associate meSNP alleles with methylation status using the variant annotations for each chromosome and each tissue in the VCF obtained after genotyping each of the animals with feed efficiency phenotypes. Of the 83 meSNPs located within 52 DMRs (3 DMRs in low feed efficiency group and 49 DMRs in high feed efficiency group) in the livers of animals with divergent feed efficiency phenotypes, 15 had genotypes called; of the 260 meSNPs in 97 DMRs (3 DMRs in low feed efficiency group and 94 DMRs in high feed efficiency group) in *longissimus dorsi* from animals with divergent feed efficiency phenotypes, 36 had genotypes called; and of the 1,103 meSNPs in 793 DMRs (296 DMRs in low feed efficiency group and 497 DMRs in high feed efficiency group) in small intestines from animals with divergent feed efficiency phenotypes, 174 had genotypes called.

After the allele frequency at each meSNP was estimated in each of the high and low feed efficiency groups, we identified 12 meSNPs with genotype call frequencies ≥ 0.5, 1 in *longissimus dorsi* and 11 in small intestine. We then identified how many of the 12 meSNPs had DNA methylation signals compatible with the alteration caused at the CpG site, that is, a meSNP allele that creates a CpG site (methylation site creating allele, or MSC allele) should be at a higher frequency in the hypermethylated group; and a meSNP allele that destroys a CpG sites (methylation site destroying allele, or MSD allele) should be at a higher frequency in the hypomethylated group. Seven meSNPs with genotype call frequencies ≥ 0.5, were identified with compatible methylation and meSNP allele patterns (all in the small intestine). When comparing, the disruption caused by these meSNPs in the CpG site as annotated in dbmeSNP, we observed that 4 had MSD and 3 had MSC alleles relative to the UMD3.1 reference assembly.

Of the 12 identified meSNPs, 3 were within the coding regions of *KIAA1549L*, *BICC1* and *WNT5B*; and 2 were located in introns of *CD226* and *ME3*. However, only the meSNPs within *KIAA1549L* and *BICC1* had DNA methylation signals that were compatible with the alleles responsible for alterations at the CpG site. None of these meSNPs are within previously identified QTL regions associated with production or feed conversion efficiency, which is not unexpected considering that the SNPs utilized in our study are novel polymorphisms identified in the 1000BGP. In both tissues, meSNPs caused a change in the genomic sequence potentially associated with differences in feed efficiency, as described in Table 3.

**Table 3:** meSNPs responsible for changes in genomic sequence associated with variation in feed efficiency in cattle.

**meSNPs with DNA methylation signal compatible with the alteration caused at the CpG site**										
**Tissue**	**Chr**	**rs number**	**Ref**	**Alt**	**meSNP**	**Methylation peaks at:**	**Allele frequency** **for HFEG**	**Allele frequency** **for LFEG**	**Region**	**Genome** **Browser**
SI	8	rs209651975	C	T	Destroying CpG	High feed efficiency	0	0.5	Intergenic	—
SI	13	rs385282371	C	G	Creating CpG	High feed efficiency	0.5	0	Intergenic	—
SI	15	rs41776554	T	C	Creating CpG	High feed efficiency	0.875	0.125	Coding	KIAA1549L
SI	19	rs209283171	G	A	Destroying CpG	Low feed efficiency	0.667	0.167	Proximal promoter	—
SI	21	rs110952331	C	T	Destroying CpG	Low feed efficiency	1	0.25	Intergenic	—
SI	26	rs110491592	C	G	Destroying CpG	Low feed efficiency	0.5	0	Intergenic	—
SI	28	rs209796881	C	G	Creating CpG	Low feed efficiency	0	0.5	3’UTR	BICC1
**meSNPs with DNA methylation signal incompatible with the alteration caused at the CpG site**										
**Tissue**	**Chr**	**rs number**	**Ref**	**Alt**	**meSNP**	**Methylation peaks at:**	**Allele frequency** **for HFEG**	**Allele frequency** **for LFEG**	**Region**	**Genome** **Browser**
SI	5	rs137028580	A	G	Creating CpG	Low feed efficiency	0.5	0	Coding	WNT5B
LD	15	rs42963062	C	T	Destroying CpG	High feed efficiency	0.75	0.25	Intergenic	—
SI	21	rs211094916	G	A	Destroying CpG	Low feed efficiency	0	0.5	Intergenic	—
SI	24	rs380531399	A	G	Creating CpG	Low feed efficiency	0.625	0	Intronic	CD226
SI	29	rs42161551	A	G	Creating CpG	High feed efficiency	0.125	0.625	Intronic	ME3

^Chr^ Chromosome

^Ref^ Reference

^Alt^ Alternative

^HFEG^ High Feed Efficiency Group

^LFEG^ Low Feed Efficiency Group

^SI^ Small intestine

^LD^ *Longissimus dorsi*

## Discussion

Methylation patterns are established and maintained at CpG dinucleotide sites [33], thus, variants caused by SNPs in regions subject to DNA methylation may alter the epigenetic profile of these regions, leading to a possible alteration in the expression of nearby genes. For this reason, it has been suggested that the relationship between DNA methylation and gene expression levels depends on the genomic context at CpG sites [10,34]. We have demonstrated that a considerable portion of the variants identified in the 1000BGP could affect epigenetic control, exerted by DNA methylation, at CpG sites. We predict that ~23% of the SNPs detected in Run5 of the 1000BGP, eliminate, create or displace these sites. An altered methylation state as a consequence of the disruption of CpG sites by SNPs has previously been reported [14] in human leukocytes where the authors found that genetic variants that disrupt DNA methylation tend to be located in genomic regions under lower selective pressure. Surprisingly, SNPs can also influence the methylation of nearby CpG sites. The presence of a SNP at a CpG site was positively correlated with the methylation of nearby CpGs in human B lymphocyte cell lines [15], suggesting that methylation profile of the region surrounding a meSNP could also be affected.

Most of the meSNPs identified in Run5 of the 1000BGP were located in intergenic regions (68%), which could harbor methylation controlled regulatory regions, and the meSNPs positioned within CGIs (~12%) were primarily found in intergenic and intronic regions. Likewise, a study in humans has found that, despite employing a technique that is also biased towards CGI, only 21.9% of CpGs that are disrupted by SNPs are found within CGIs suggesting that SNPs that disrupt CpGs tend to be located in intergenic regions that are not CGIs [14], corroborating our observations.

Several authors have reported the impact of SNPs located within coding regions and/or promoters on DNA methylation profiles, gene transcription and function [9,10,13,35–37]. This impact occurs due to changes in protein sequence or in cis-regulatory elements [38]. However, when these markers are located outside of functionally annotated regions, as found in our study, it becomes more difficult to predict their function within the context of gene expression and, by consequence, effects on phenotypes.

The second largest number of meSNPs was found within intronic regions (~26%). Besides affecting downstream and/or distal regulatory regions, such as enhancers, SNPs in intronic regions may play an important role in regulating global methylation patterns, as previously shown in human lymphoblastoid cells where a genome-wide significant association between *rs10876043*, located within an intron of *DIP2B*, with methylation patterns was demonstrated [13]. Further, the authors also reported that SNPs associated with methylation were also enriched for association with nearby (2 Kb) CpG sites [13,15], indicating that the presence of a SNP at a CpG can alter the epigenetic regulation of the region by also affecting the methylation of nearby CpGs.

In addition to determining the genomic distribution for each class of meSNPs, we also predicted functional annotations to create the largest database of annotated meSNPs produced in cattle to date. This database, dbmeSNP, provides valuable information to researchers investigating the role of SNPs as possible regulators of gene expression, or influencing phenotypic associations.

In the bovine, the additive effects of SNPs frequently explains from 32 to 80% of the genetic variation, depending on the phenotype [39,40], however, just how much of the variation is due to differences in methylation state is difficult to elucidate [36]. In an application of dbmeSNP, we determined which meSNPs were located within CGIs, using NCBI bovine CGI data, and then attempted to identify those within MIRA-Seq determined DMRs found within bovine tissues from high and low feed efficiency animals.

We first determined whether there was an enrichment of meSNPs within CGIs and DMRs from tissues assayed by MIRA-Seq, which would suggest a potential role for these methyl-markers in the regulation of methylation patterns in these DMRs. By comparing the observed and expected number of meSNPs within CGIs, based upon the assumption of a random distribution throughout the genome, we found a 2-fold enrichment of meSNPs within CGIs (p<0.01), most within intergenic regions. Based on the premise that genetic variants at CpG sites can disrupt methylation and change the methylation profile of the region [14,15], this enrichment suggests that these meSNPs, whether inserting or deleting CpG sites, could affect methylation patterns in CGIs, which may alter nearby gene expression and, consequently, influence phenotypes. Approximately 0.7% of all identified meSNPs were mapped to CGIs within DMRs found between animals with a high or low feed efficiency phenotype. We also observed approximately a 2-fold enrichment of meSNPs within CGIs relative to the genome as a whole, which reinforces the likelihood that meSNPs may alter methylation patterns and regulate gene expression resulting in phenotypic variation. Next, we associated meSNPs mapped to the DMRs of animals that differed in feed efficiency with the genotypes (VCF containing variants) for each chromosome and each evaluated tissue. We found 12 meSNPs that caused a change in the genomic sequence within DMRs associated with feed efficiency phenotype, and 7 of the meSNPs had allelic associations that were consistent with methylation patterns. Several studies have identified SNPs associated with feed efficiency in cattle, through the use of the Illumina BovineSNP50 v3 BeadChip [41–43] that has a genomic coverage of 53,714 markers. We chose to perform a genome-wide analysis with the 56,969,697 annotated SNPs identified in Run5 of the 1000BGP, with the purpose of significantly increasing the chance of identifying meSNPs within DMRs between the high and low feed efficiency animals, and for which allelic associations were concordant with the methylation profile of the feed efficiency groups.

Sequence-based methods present a unique opportunity to assign epigenetic marks to specific alleles. Previous work identified SNPs within 1,000 human embryonic stem cell CGIs that overlapped between MRE-Seq and MeDIP-Seq signals. Of 1,000 examined CGI loci, 203 contained an informative SNP and 31% of these exhibited concordant allelic associations, in which the allele responsible for creating a CpG motif was associated with hypermethylation [44]. We observed that of the 7 meSNPs in DMRs of tissues from animals differing in feed efficiency, with DNA methylation signal compatible with the alteration caused at the CpG site, 2 were found in protein coding regions, one creating a synonymous substitution in *KIAA1549L* and the other in the 3' UTR of *BICC1*, both creating a new CpG site in each DMR. Thus, these 2 meSNPs were established as candidates for influencing methylation patterns in these regions via alterations in the regulation of gene expression.

In addition to sequence-based methods, epigenome-wide association studies (EWAS) have been conducted for a wide range of diseases including mental health conditions, cardiovascular disease and cancers [45]. Clear links have been established between abnormal DNA methylation and human diseases [46], such as lung cancer [47], general mental health [48,49] and diabetes [9,50,51]. There is also a considerable interest in identifying potential biomarkers, such as CpG sites, for use in algorithms designed to predict risk of disease. By evaluating leukocyte DNA methylation patterns to identify potential biomarkers for the early detection of colorectal cancer, two differentially methylated CpG sites located in the promoter region of *KIAA1549L* were identified. Logistic regression models were then used to predict risk colon cancer based on promoter methylation status with accuracies of 0.73 in clinical settings, and 0.69 in screenings [52]. We identified a meSNP associated with the feed efficiency phenotype that creates a CpG site in the coding region of *KIAA1549L* and while the function of this gene is largely uncharacterized [52], methylation patterns appear to regulate the expression of this gene suggesting that it should be further examined for effects on feed efficiency. The relationship between gene methylation and the level of gene expression is complex. While methylation in the promoter region of genes can block the initiation of transcription, the role of DNA methylation within the gene body on gene expression is not well understood [53].

There are recent genome-wide association studies (GWAS) that have associated *BICC1* with depression [54,55], bone mineral density [56,57] and muscle mass [58]. Of particular interest, epigenetic regulation of *BICC1* seems to be a biomarker for late-onset hypertrophy in human skeletal muscle, with acute and sustained hypomethylation regulating gene expression and hypertrophy [58]. The effect of epigenetic regulation of *BICC1* on muscle hypotrophy suggests a potential role for this gene on feed efficiency which is a function of both feed intake and body growth.

A limitation of this study is that MIRA-Seq only captures short CGIs where substantial methylation occurs, not providing information about CGIs with low levels of methylation. Therefore, when comparing methylation profiles of DNA extracted from tissues from cattle with divergent feed efficiencies, a CGI that is methylated in one group but not the other, cannot be analyzed because there are no variant calls for the second group. Despite this, we were able to locate a few of our identified meSNPs within DMRs in tissues from animals varying in feed efficiency, indicating that our developed dbmeSNP is useful for correlating meSNPs and methylation profiles that differ between phenotypes.

We suggest that meSNPs may mechanistically induce a quantitative difference in methylation between high and low feed efficiency animals through the creation or destruction of CpG sites. Our dbmeSNP database can facilitate the identification of functional "epigenetic polymorphisms" underlying variation in any bovine phenotype.

## Supporting information

S1 Table Table containing the classification of meSNPs into 13 different categories based on the 35 sequence ontology (SO term) annotations provided by Ensembl. (PDF)

S2 Table MIRA-Seq profile datasets and sample description of tissues from cattle with divergent feed efficiencies. (PDF)

S3 Table Table containing the estimated enrichment of meSNPs in CpG islands, by chromosome, using the Chi-square test. ^Chr^ Chromosome ^bp^ Base pairs ^CGIs^ CpG islands.
(PDF)

S4 Table Table containing the estimated enrichment of meSNPs in DMRs in the tissues submitted to MIRA-Seq, by chromosome, using the Chi-square test. ^Chr^ Chromosome ^bp^ Base pairs ^AE^ Average enrichment.
(PDF)
