# Supplementary material for: Identification of bovine CpG SNPs as potential targets for epigenetic regulation via DNA methylation
Source: PLoS One. 2019 Sep 12;14(9):e0222329. doi: 10.1371/journal.pone.0222329 (PMC6742455; doi:10.1371/journal.pone.0222329)
Supplement: S1 Table — (PDF) [file pone.0222329.s001.pdf]

S1 Table. Classification of meSNPs from the sequence ontology (SO term) annotation provided by Ensembl

Categories	Variables that make up each category (SO term)
Proximal promoter	Upstream gene variant
Intronic region	Splice acceptor variant
	Splice donor variant
	Intron variant
Coding region	Stop gained
	Frameshift variant
	Stop lost
	Start lost
	Inframe insertion
	Inframe deletion
	Missense variant
	Protein altering variant
	Incomplete terminal codon variant
	Stop retained variant
	Synonymous variant
	Coding sequence variant
	NMD transcript variant
Non-coding RNA region	Mature miRNA variant
	Non-coding transcript exon variant
	Non coding transcript variant
Intergenic region	Downstream gene variant
	Intergenic variant
Regulatory region	TFBS ablation
	TFBS amplification
	TF binding site variant
	Regulatory region ablation
	Regulatory region amplification
	Regulatory region variant
5’UTR	5 prime UTR variant
3’UTR	3 prime UTR variant
Transcript deletion region	Transcript ablation
Transcription amplification region	Transcript amplification
Splice region	Splice region variant
Elongation region	Feature elongation
Feature truncation	Feature truncation
