# Supplementary material for: Identification of bovine CpG SNPs as potential targets for epigenetic regulation via DNA methylation
Source: PLoS One. 2019 Sep 12;14(9):e0222329. doi: 10.1371/journal.pone.0222329 (PMC6742455; doi:10.1371/journal.pone.0222329)
Supplement: S2 Table — (PDF) [file pone.0222329.s002.pdf]

S2 Table. MIRA-Seq profile datasets and sample description of tissues from cattle with divergent feed efficiencies

Sample ID	Tissue type	Feed efficiency	SRA_run ID	Raw file name	Processed file name
LVR01	Liver	High	SRR9961549	AN.2482.3888.I.AS.01.1.fastq	LVR01_peaks.narrowPeak
LVR02	Liver	High	SRR9961532	AN.2921.3872.I.CS.01.1.fastq	LVR02_peaks.narrowPeak
LVR03	Liver	High	SRR9961560	AN.1692.3920.I.AS.01.1.fastq	LVR03_peaks.narrowPeak
LVR04	Liver	High	SRR9961565	AN.1688.3856.I.BS.01.1.fastq	LVR04_peaks.narrowPeak
LVR05	Liver	Low	SRR9961539	AN.2955.3968.I.AS.01.1.fastq	LVR05_peaks.narrowPeak
LVR06	Liver	Low	SRR9961554	AN.2512.4064.I.BS.01.1.fastq	LVR06_peaks.narrowPeak
LVR07	Liver	Low	SRR9961541	AN.2759.56.I.AS.01.1.fastq	LVR07_peaks.narrowPeak
LVR08	Liver	Low	SRR9961546	AN.2933.3984.I.AS.01.1.fastq	LVR08_peaks.narrowPeak
REM01	Ribeye Muscle	High	SRR9961548	AN.2482.3885.I.AS.01.1.fastq	REM01_peaks.narrowPeak
REM02	Ribeye Muscle	High	SRR9961535	AN.2921.3869.I.AS.01.1.fastq	REM02_peaks.narrowPeak
REM03	Ribeye Muscle	High	SRR9961559	AN.1692.3917.I.AS.01.1.fastq	REM03_peaks.narrowPeak
REM04	Ribeye Muscle	High	SRR9961563	AN.1688.3853.I.AS.01.1.fastq	REM04_peaks.narrowPeak
REM05	Ribeye Muscle	Low	SRR9961543	AN.2955.3965.I.BS.01.1.fastq	REM05_peaks.narrowPeak
REM06	Ribeye Muscle	Low	SRR9961552	AN.2512.4061.I.BS.01.1.fastq	REM06_peaks.narrowPeak
REM07	Ribeye Muscle	Low	SRR9961540	AN.2759.53.I.AS.01.1.fastq	REM07_peaks.narrowPeak
REM08	Ribeye Muscle	Low	SRR9961542	AN.2955.3965.I.BS.01.1.fastq	REM08_peaks.narrowPeak
SI01	Small Intestine	High	SRR9961557	AN.2482.3882.I.AS.01.1.fastq	SI01_peaks.narrowPeak
SI02	Small Intestine	High	SRR9961536	AN.2921.3866.I.BS.01.1.fastq	SI02_peaks.narrowPeak
SI03	Small Intestine	High	SRR9961558	AN.1692.3914.I.AS.01.1.fastq	SI03_peaks.narrowPeak
SI04	Small Intestine	High	SRR9961562	AN.1688.3850.I.AS.01.1.fastq	SI04_peaks.narrowPeak
SI05	Small Intestine	Low	SRR9961544	AN.2955.3962.I.AS.01.1.fastq	SI05_peaks.narrowPeak
SI06	Small Intestine	Low	SRR9961550	AN.2512.4058.I.AS.01.1.fastq	SI06_peaks.narrowPeak
SI07	Small Intestine	Low	SRR9961555	AN.2759.50.I.AS.01.1.fastq	SI07_peaks.narrowPeak
SI08	Small Intestine	Low	SRR9961537	AN.2933.3978.I.BS.01.1.fastq	SI08_peaks.narrowPeak
