# Supplementary material for: Identification of bovine CpG SNPs as potential targets for epigenetic regulation via DNA methylation
Source: PLoS One. 2019 Sep 12;14(9):e0222329. doi: 10.1371/journal.pone.0222329 (PMC6742455; doi:10.1371/journal.pone.0222329)
Supplement: S3 Table — Chr Chromosome bp Base pairs CGIs CpG islands. (PDF) [file pone.0222329.s003.pdf]

S3 Table. Estimation of the enrichment of meSNPs in relaxed and strict CGIs by Chi-square test

Chr	Quantity of meSNPs in the database	bp in CGIs	Chr lenght	(bp/Chr lenght)	Expected quantity of meSNPs	Observed quantity of meSNPs	P-value	Enrichment
Relaxed CGIs								
1	710562	4067505	158337067	0,0257	18254	50386	0,00E+00	2,76
2	623534	4278138	137060424	0,0312	19463	52508	0,00E+00	2,70
3	551403	4620427	121430405	0,0381	20981	53945	0,00E+00	2,57
4	570575	4250696	120829699	0,0352	20072	52517	0,00E+00	2,62
5	566935	4991116	121191424	0,0412	23349	58389	0,00E+00	2,50
6	527642	3753120	119458736	0,0314	16577	45406	0,00E+00	2,74
7	503117	4491933	112638659	0,0399	20064	47065	0,00E+00	2,35
8	506959	3851578	113384836	0,0340	17221	45373	0,00E+00	2,63
9	475586	3521317	105708250	0,0333	15843	47077	0,00E+00	2,97
10	484571	3479523	104305016	0,0334	16165	41654	0,00E+00	2,58
11	515527	4953534	107310763	0,0462	23797	59271	0,00E+00	2,49
12	506143	3389635	91163125	0,0372	18819	44811	0,00E+00	2,38
13	430866	3968773	84240350	0,0471	20299	46969	0,00E+00	2,31
14	414976	3192460	84648390	0,0377	15651	42020	0,00E+00	2,68
15	438984	2965363	85296676	0,0348	15261	38450	0,00E+00	2,52
16	424372	3523405	81724687	0,0431	18296	43487	0,00E+00	2,38
17	374176	3740666	75158596	0,0498	18623	43153	0,00E+00	2,32
18	353610	4805445	66004023	0,0728	25745	51040	0,00E+00	1,98
19	344415	4871786	64057457	0,0761	26194	51814	0,00E+00	1,98
20	350451	2487534	72042655	0,0345	12101	31778	0,00E+00	2,63
21	371682	3464824	71599096	0,0484	17986	42513	0,00E+00	2,36
22	306917	3484748	61435874	0,0567	17409	41210	0,00E+00	2,37
23	351291	3389236	52530062	0,0645	22665	47656	0,00E+00	2,10
24	334277	2985030	62714930	0,0476	15911	38240	0,00E+00	2,40
25	267955	4275434	42904170	0,0997	26702	49157	0,00E+00	1,84
26	264034	2588837	51681464	0,0501	13226	32560	0,00E+00	2,46
27	247459	2599870	45407902	0,0573	14168	32810	0,00E+00	2,32
28	249140	1525166	46312546	0,0329	8205	20847	0,00E+00	2,54
29	313983	3191183	51505224	0,0620	19454	40902	0,00E+00	2,10
X	455621	3551511	148823899	0,0239	10873	37062	0,00E+00	3,41
Strict CGIs								
1	710562	976964	158337067	0,0062	4384	8127	0,00E+00	1,85
2	623534	1078846	137060424	0,0079	4908	9358	0,00E+00	1,91
3	551403	1314876	121430405	0,0108	5971	10997	0,00E+00	1,84
4	570575	1090660	120829699	0,0090	5150	10679	0,00E+00	2,07
5	566935	1196345	121191424	0,0099	5597	10191	0,00E+00	1,82
6	527642	924648	119458736	0,0077	4084	8085	0,00E+00	1,98
7	503117	1412035	112638659	0,0125	6307	10957	0,00E+00	1,74
8	506959	1184482	113384836	0,0104	5296	10589	0,00E+00	2,00
9	475586	841833	105708250	0,0080	3787	9478	0,00E+00	2,50
10	484571	981421	104305016	0,0094	4559	8846	0,00E+00	1,94
11	515527	1253721	107310763	0,0117	6023	11541	0,00E+00	1,92
12	506143	667846	91163125	0,0073	3708	7172	0,00E+00	1,93
13	430866	910836	84240350	0,0108	4659	7654	0,00E+00	1,64
14	414976	728558	84648390	0,0086	3572	7563	0,00E+00	2,12

15	438984	678844	85296676	0,0080	3494	6501	0,00E+00	1,86
16	424372	809019	81724687	0,0099	4201	7346	0,00E+00	1,75
17	374176	888739	75158596	0,0118	4425	8406	0,00E+00	1,90
18	353610	1396524	66004023	0,0212	7482	11114	0,00E+00	1,49
19	344415	1408591	64057457	0,0220	7574	11535	0,00E+00	1,52
20	350451	526202	72042655	0,0073	2560	4967	0,00E+00	1,94
21	371682	825248	71599096	0,0115	4284	7902	0,00E+00	1,84
22	306917	782390	61435874	0,0127	3909	7303	0,00E+00	1,87
23	351291	755770	52530062	0,0144	5054	9448	0,00E+00	1,87
24	334277	640311	62714930	0,0102	3413	6706	0,00E+00	1,96
25	267955	1264133	42904170	0,0295	7895	11502	0,00E+00	1,46
26	264034	595091	51681464	0,0115	3040	5966	0,00E+00	1,96
27	247459	608849	45407902	0,0134	3318	6292	0,00E+00	1,90
28	249140	412694	46312546	0,0089	2220	4550	0,00E+00	2,05
29	313983	739181	51505224	0,0144	4506	7506	0,00E+00	1,67
X	455621	952293	148823899	0,0064	2915	7615	0,00E+00	2,61

[Chr] Chromosome
[bp] Base pairs
[CGIs] CpG islands
