# Supplementary material for: Identification of bovine CpG SNPs as potential targets for epigenetic regulation via DNA methylation
Source: PLoS One. 2019 Sep 12;14(9):e0222329. doi: 10.1371/journal.pone.0222329 (PMC6742455; doi:10.1371/journal.pone.0222329)
Supplement: S4 Table — Chr Chromosome bp Base pairs AE Average enrichment. (PDF) [file pone.0222329.s004.pdf]

S4 Table. Estimation of the enrichment of meSNPs in tissues submitted to MIRA-Seq by Chi-square test

Chr	Quantity of meSNPs in the database	bp in the methylated regions	Chr lenght	(bp / Chr lenght)	Expected quantity of meSNPs	Observed quantity of meSNPs	P-value	Enrichment
				**LIVER - High feed efficiency animals**				
1	710562	244408	158337067	0,0015	1097	3353	0,00E+00	3,06
2	623534	221121	137060424	0,0016	1006	3087	0,00E+00	3,07
3	551403	211970	121430405	0,0017	963	2693	0,00E+00	2,80
4	570575	196860	120829699	0,0016	930	2749	0,00E+00	2,96
5	566935	243639	121191424	0,0020	1140	3099	0,00E+00	2,72
6	527642	156371	119458736	0,0013	691	2257	0,00E+00	3,27
7	503117	283299	112638659	0,0025	1265	3645	0,00E+00	2,88
8	506959	194761	113384836	0,0017	871	2637	0,00E+00	3,03
9	475586	177602	105708250	0,0017	799	2610	0,00E+00	3,27
10	484571	214106	104305016	0,0021	995	2822	0,00E+00	2,84
11	515527	250554	107310763	0,0023	1204	3210	0,00E+00	2,67
12	506143	176735	91163125	0,0019	981	2353	0,00E+00	2,40
13	430866	237540	84240350	0,0028	1215	3068	0,00E+00	2,53
14	414976	213756	84648390	0,0025	1048	3352	0,00E+00	3,20
15	438984	184606	85296676	0,0022	950	2674	0,00E+00	2,81
16	424372	197533	81724687	0,0024	1026	2738	0,00E+00	2,67
17	374176	189531	75158596	0,0025	944	2422	0,00E+00	2,57
18	353610	235846	66004023	0,0036	1264	2626	0,00E+00	2,08
19	344415	215587	64057457	0,0034	1159	2563	0,00E+00	2,21
20	350451	138953	72042655	0,0019	676	1983	0,00E+00	2,93
21	371682	165987	71599096	0,0023	862	2166	0,00E+00	2,51
22	306917	161732	61435874	0,0026	808	1954	0,00E+00	2,42
23	351291	139126	52530062	0,0026	930	2251	0,00E+00	2,42
24	334277	186350	62714930	0,0030	993	2561	0,00E+00	2,58
25	267955	148253	42904170	0,0035	926	1864	1,06E-208	2,01
26	264034	124791	51681464	0,0024	638	1665	0,00E+00	2,61
27	247459	154334	45407902	0,0034	841	2540	0,00E+00	3,02
28	249140	101184	46312546	0,0022	544	1530	0,00E+00	2,81
29	313983	128822	51505224	0,0025	785	1986	0,00E+00	2,53
X	455621	97409	148823899	0,0007	298	1823	0,00E+00	6,11
								**AE = 2,83**
				**LIVER - Low feed efficiency animals**				
1	710562	27740	158337067	0,0002	124	379	3,56E-115	3,04
2	623534	20938	137060424	0,0002	95	372	7,13E-177	3,91
3	551403	27289	121430405	0,0002	124	438	3,83E-175	3,53
4	570575	21038	120829699	0,0002	99	334	1,49E-122	3,36
5	566935	21993	121191424	0,0002	103	387	1,20E-172	3,76
6	527642	19053	119458736	0,0002	84	238	4,03E-63	2,83
7	503117	34662	112638659	0,0003	155	524	1,87E-193	3,38
8	506959	26638	113384836	0,0002	119	425	7,08E-173	3,57
9	475586	23521	105708250	0,0002	106	428	2,57E-215	4,04
10	484571	18284	104305016	0,0002	85	228	2,46E-54	2,68

11	515527	34012	107310763	0,0003	163	514	1,26E-165	3,15
12	506143	17704	91163125	0,0002	98	264	1,04E-62	2,69
13	430866	20711	84240350	0,0002	106	304	1,57E-82	2,87
14	414976	34556	84648390	0,0004	169	705	0,00E+00	4,16
15	438984	17569	85296676	0,0002	90	331	3,16E-141	3,66
16	424372	22779	81724687	0,0003	118	258	9,01E-38	2,18
17	374176	28977	75158596	0,0004	144	331	1,66E-54	2,29
18	353610	20827	66004023	0,0003	112	237	1,62E-32	2,12
19	344415	18547	64057457	0,0003	100	341	5,63E-129	3,42
20	350451	17305	72042655	0,0002	84	258	4,85E-80	3,06
21	371682	16254	71599096	0,0002	84	218	6,12E-48	2,58
22	306917	11132	61435874	0,0002	56	121	1,82E-18	2,18
23	351291	15639	52530062	0,0003	105	283	3,69E-68	2,71
24	334277	18262	62714930	0,0003	97	321	8,88E-114	3,30
25	267955	16140	42904170	0,0004	101	306	7,66E-93	3,04
26	264034	11581	51681464	0,0002	59	188	5,73E-63	3,18
27	247459	19197	45407902	0,0004	105	386	1,32E-166	3,69
28	249140	14770	46312546	0,0003	79	258	3,01E-89	3,25
29	313983	14457	51505224	0,0003	88	286	1,29E-98	3,25
X	455621	18893	148823899	0,0001	58	566	0,00E+00	9,79
								**AE = 3,36**
				**RIBEYE - High feed efficiency animals**				
1	710562	334007	158337067	0,0021	1499	4854	0,00E+00	3,24
2	623534	314607	137060424	0,0023	1431	4329	0,00E+00	3,02
3	551403	302933	121430405	0,0025	1376	3961	0,00E+00	2,88
4	570575	303680	120829699	0,0025	1434	4084	0,00E+00	2,85
5	566935	332278	121191424	0,0027	1554	4307	0,00E+00	2,77
6	527642	234991	119458736	0,0020	1038	3267	0,00E+00	3,15
7	503117	425354	112638659	0,0038	1900	5183	0,00E+00	2,73
8	506959	290682	113384836	0,0026	1300	4323	0,00E+00	3,33
9	475586	273289	105708250	0,0026	1230	4277	0,00E+00	3,48
10	484571	300597	104305016	0,0029	1396	4288	0,00E+00	3,07
11	515527	365345	107310763	0,0034	1755	4873	0,00E+00	2,78
12	506143	247939	91163125	0,0027	1377	3421	0,00E+00	2,49
13	430866	359398	84240350	0,0043	1838	4895	0,00E+00	2,66
14	414976	294061	84648390	0,0035	1442	4577	0,00E+00	3,17
15	438984	252125	85296676	0,0030	1298	3636	0,00E+00	2,80
16	424372	301487	81724687	0,0037	1566	4184	0,00E+00	2,67
17	374176	284508	75158596	0,0038	1416	3550	0,00E+00	2,51
18	353610	385772	66004023	0,0058	2067	4372	0,00E+00	2,12
19	344415	342692	64057457	0,0053	1843	4318	0,00E+00	2,34
20	350451	195838	72042655	0,0027	953	2931	0,00E+00	3,08
21	371682	275968	71599096	0,0039	1433	3638	0,00E+00	2,54
22	306917	233163	61435874	0,0038	1165	2733	0,00E+00	2,35
23	351291	215171	52530062	0,0041	1439	3443	0,00E+00	2,39
24	334277	282796	62714930	0,0045	1507	4067	0,00E+00	2,70
25	267955	237436	42904170	0,0055	1483	3125	0,00E+00	2,11
26	264034	184192	51681464	0,0036	941	2679	0,00E+00	2,85
27	247459	220708	45407902	0,0049	1203	3330	0,00E+00	2,77

28	249140	144788	46312546	0,0031	779	2290	0,00E+00	2,94
29	313983	206437	51505224	0,0040	1258	2986	0,00E+00	2,37
X	455621	153017	148823899	0,0010	468	2727	0,00E+00	5,82
								**AE = 2,87**
**RIBEYE - Low feed efficiency animals**								
1	710562	73693	158337067	0,0005	331	853	2,12E-181	2,58
2	623534	70255	137060424	0,0005	320	958	2,92E-279	3,00
3	551403	67271	121430405	0,0006	305	788	8,86E-168	2,58
4	570575	77585	120829699	0,0006	366	859	4,46E-146	2,34
5	566935	70827	121191424	0,0006	331	785	4,13E-137	2,37
6	527642	63336	119458736	0,0005	280	651	3,74E-109	2,33
7	503117	76970	112638659	0,0007	344	950	1,91E-234	2,76
8	506959	65814	113384836	0,0006	294	892	5,05E-266	3,03
9	475586	56959	105708250	0,0005	256	1034	0,00E+00	4,03
10	484571	57546	104305016	0,0006	267	791	4,62E-225	2,96
11	515527	83723	107310763	0,0008	402	1198	0,00E+00	2,98
12	506143	51001	91163125	0,0006	283	564	1,56E-62	1,99
13	430866	73668	84240350	0,0009	377	1017	1,49E-238	2,70
14	414976	64462	84648390	0,0008	316	1111	0,00E+00	3,52
15	438984	47788	85296676	0,0006	246	742	1,38E-219	3,02
16	424372	57696	81724687	0,0007	300	769	5,86E-162	2,57
17	374176	61609	75158596	0,0008	307	802	6,08E-176	2,61
18	353610	57695	66004023	0,0009	309	610	1,14E-65	1,97
19	344415	61081	64057457	0,0010	328	773	6,59E-133	2,35
20	350451	42667	72042655	0,0006	208	556	3,09E-129	2,68
21	371682	47876	71599096	0,0007	249	590	4,89E-104	2,37
22	306917	53505	61435874	0,0009	267	498	3,25E-45	1,86
23	351291	43522	52530062	0,0008	291	871	2,70E-253	2,99
24	334277	46174	62714930	0,0007	246	615	2,93E-122	2,50
25	267955	47656	42904170	0,0011	298	775	1,59E-168	2,60
26	264034	41392	51681464	0,0008	211	513	1,66E-95	2,43
27	247459	45109	45407902	0,0010	246	678	3,03E-167	2,76
28	249140	37018	46312546	0,0008	199	515	5,78E-111	2,59
29	313983	40102	51505224	0,0008	244	626	1,64E-131	2,56
X	455621	34141	148823899	0,0002	105	786	0,00E+00	7,52
								**AE = 2,82**
**SMALL INTESTINE - High feed efficiency animals**								
1	710562	359980	158337067	0,0023	1615	4716	0,00E+00	2,92
2	623534	316126	137060424	0,0023	1438	4008	0,00E+00	2,79
3	551403	346225	121430405	0,0029	1572	4205	0,00E+00	2,67
4	570575	302803	120829699	0,0025	1430	4068	0,00E+00	2,84
5	566935	345314	121191424	0,0028	1615	4247	0,00E+00	2,63
6	527642	239080	119458736	0,0020	1056	3247	0,00E+00	3,07
7	503117	428848	112638659	0,0038	1916	5215	0,00E+00	2,72
8	506959	325461	113384836	0,0029	1455	4324	0,00E+00	2,97
9	475586	278785	105708250	0,0026	1254	3977	0,00E+00	3,17
10	484571	342787	104305016	0,0033	1592	4213	0,00E+00	2,65
11	515527	370336	107310763	0,0035	1779	4204	0,00E+00	2,36
12	506143	271832	91163125	0,0030	1509	3552	0,00E+00	2,35

13	430866	403755	84240350	0,0048	2065	4995	0,00E+00	2,42
14	414976	319440	84648390	0,0038	1566	4771	0,00E+00	3,05
15	438984	286922	85296676	0,0034	1477	4004	0,00E+00	2,71
16	424372	328553	81724687	0,0040	1706	4313	0,00E+00	2,53
17	374176	300793	75158596	0,0040	1497	3593	0,00E+00	2,40
18	353610	379057	66004023	0,0057	2031	4320	0,00E+00	2,13
19	344415	353089	64057457	0,0055	1898	4120	0,00E+00	2,17
20	350451	210372	72042655	0,0029	1023	2672	0,00E+00	2,61
21	371682	282020	71599096	0,0039	1464	3567	0,00E+00	2,44
22	306917	250974	61435874	0,0041	1254	3009	0,00E+00	2,40
23	351291	220485	52530062	0,0042	1474	3299	0,00E+00	2,24
24	334277	302654	62714930	0,0048	1613	4172	0,00E+00	2,59
25	267955	222529	42904170	0,0052	1390	2686	6,98E-265	1,93
26	264034	193016	51681464	0,0037	986	2700	0,00E+00	2,74
27	247459	244603	45407902	0,0054	1333	3418	0,00E+00	2,56
28	249140	174562	46312546	0,0038	939	2546	0,00E+00	2,71
29	313983	211032	51505224	0,0041	1286	2962	0,00E+00	2,30
X	455621	125212	148823899	0,0008	383	2503	0,00E+00	6,53
								**AE = 2,72**
			**SMALL INTESTINE - Low feed efficiency animals**					
1	710562	274398	158337067	0,0017	1231	3907	0,00E+00	3,17
2	623534	257922	137060424	0,0019	1173	3541	0,00E+00	3,02
3	551403	263950	121430405	0,0022	1199	3438	0,00E+00	2,87
4	570575	245396	120829699	0,0020	1159	3353	0,00E+00	2,89
5	566935	260634	121191424	0,0022	1219	3449	0,00E+00	2,83
6	527642	180256	119458736	0,0015	796	2558	0,00E+00	3,21
7	503117	324176	112638659	0,0029	1448	4052	0,00E+00	2,80
8	506959	252294	113384836	0,0022	1128	3551	0,00E+00	3,15
9	475586	215340	105708250	0,0020	969	3288	0,00E+00	3,39
10	484571	238998	104305016	0,0023	1110	3333	0,00E+00	3,00
11	515527	288631	107310763	0,0027	1387	3812	0,00E+00	2,75
12	506143	213646	91163125	0,0023	1186	3197	0,00E+00	2,70
13	430866	303747	84240350	0,0036	1554	3989	0,00E+00	2,57
14	414976	236740	84648390	0,0028	1161	3674	0,00E+00	3,17
15	438984	224203	85296676	0,0026	1154	3366	0,00E+00	2,92
16	424372	237545	81724687	0,0029	1234	3246	0,00E+00	2,63
17	374176	224862	75158596	0,0030	1119	2673	0,00E+00	2,39
18	353610	274766	66004023	0,0042	1472	3226	0,00E+00	2,19
19	344415	274673	64057457	0,0043	1477	3435	0,00E+00	2,33
20	350451	166149	72042655	0,0023	808	2373	0,00E+00	2,94
21	371682	213992	71599096	0,0030	1111	2764	0,00E+00	2,49
22	306917	184142	61435874	0,0030	920	2134	0,00E+00	2,32
23	351291	175238	52530062	0,0033	1172	3072	0,00E+00	2,62
24	334277	240199	62714930	0,0038	1280	3486	0,00E+00	2,72
25	267955	175710	42904170	0,0041	1097	2368	0,00E+00	2,16
26	264034	148049	51681464	0,0029	756	2179	0,00E+00	2,88
27	247459	176415	45407902	0,0039	961	2751	0,00E+00	2,86
28	249140	135785	46312546	0,0029	730	2000	0,00E+00	2,74
29	313983	149409	51505224	0,0029	911	2222	0,00E+00	2,44

X	455621	101644	148823899	0,0007	311	2043	0,00E+00	6,57
								**AE = 2,89**

[Chr] Chromosome
[bp] Base pairs
[AE] Average enrichment
